# The Influence of Three-Dimensional Impaction Depth on Surgical Difficulty During Mandibular Third Molar Surgery: A Cone-Beam Computed Tomography (CBCT)-Based Prospective Observational Study

**DOI:** 10.7759/cureus.113861

**Published:** 2026-08-03

**Authors:** Swapnil U Shinde, Bobby Joseph, Arani Roy, Kolli Giri, Jay Gohil, Abida Parveen

**Affiliations:** 1 Department of Oral and Maxillofacial Surgery, Bharati Vidyapeeth (Deemed to be University) Dental College and Hospital, Sangli, IND; 2 Department of Conservative Dentistry and Endodontics, St. Gregorios Dental College, Ernakulam, IND; 3 Department of Dentistry, Balurghat District Hospital, Balurghat, IND; 4 Department of Oral and Maxillofacial Surgery, Drs. Sudha and Nageswara Rao Siddhartha Institute of Dental Sciences, Chinaoutpalli, IND; 5 Department of Prosthodontics, K. M. Shah Dental College and Hospital, Sumandeep Vidyapeeth (Deemed to be University), Vadodara, IND; 6 Department of Orthodontics, Mahirishi Markandeshwar College of Dental Sciences and Research, Ambala, IND

**Keywords:** cone-beam computed tomography, impacted mandibular third molar, operative time, surgical difficulty, three-dimensional impaction depth index

## Abstract

Introduction

Accurate preoperative prediction of the surgical difficulty associated with impacted mandibular third molars is essential for treatment planning and the minimization of operative complications. Conventional radiographic classifications have a limited ability to accurately represent the three-dimensional position of impacted teeth. This study aimed to evaluate the influence of a novel cone-beam CT (CBCT)-derived three-dimensional impaction depth index (3D-IDI) on the surgical difficulty of mandibular third molar removal. A novel 3D-IDI was developed to integrate the vertical, buccolingual, and anteroposterior dimensions into a single quantitative measure.

Methods

This study involved 90 patients with unilaterally impacted mandibular third molars who underwent standardized preoperative CBCT imaging. Vertical, buccolingual, and anteroposterior depths were measured to calculate 3D-IDI. Surgical difficulty was assessed using operative time and surgeon-assigned difficulty score. Receiver operating characteristic (ROC) curve, correlation, and multivariable linear regression analyses were performed.

Results

The mean participant age was 27.13 ± 4.56 years, and the mean 3D-IDI was 11.04 ± 2.68 mm. ROC analysis demonstrated excellent predictive performance of the 3D-IDI for identifying surgically difficult cases (area under the curve (AUC) = 0.938; 95% confidence interval (CI): 0.888-0.988; p < 0.001), with an optimal cut-off value of 9.67 mm, sensitivity of 88.2%, specificity of 89.7%, and overall accuracy of 88.9%. Deep impactions showed significantly longer operative time (42.45 ± 4.93 vs. 30.02 ± 4.02 minutes), higher surgical difficulty scores, and more frequent tooth sectioning than shallow impactions (all p ≤ 0.002). 3D-IDI demonstrated strong positive correlations with operative time (ρ = 0.901), and surgical difficulty score (ρ = 0.860) (all p < 0.001). Vertical and anteroposterior depths were identified as independent predictors of operative time.

Conclusions

The novel CBCT-derived 3D-IDI is a reliable predictor of surgical difficulty during mandibular third molar removal. Its incorporation into preoperative assessments may facilitate objective risk stratification, improve surgical planning, and optimize patient management. Further validation in larger samples is required.

## Introduction

The extraction of impacted mandibular third molars is a cornerstone procedure in oral and maxillofacial surgery owing to the high prevalence of third molar impaction and its associated clinical indications. The degree of surgical difficulty varies considerably among patients and depends on several anatomical and radiographic factors, including the depth of impaction, angulation, root morphology, relationship with the mandibular canal, and surrounding bone [1]. Accurate preoperative assessment of these variables is essential for predicting operative complexity, minimizing complications, improving patient counseling, and facilitating appropriate surgical planning [2].

Conventional two-dimensional radiographic classifications have been widely used to estimate the difficulty of third molar surgery. However, these systems provide limited information because they cannot adequately depict the three-dimensional spatial relationship between an impacted tooth and adjacent anatomical structures [3,4]. Cone-beam CT (CBCT) overcomes these limitations by providing high-resolution three-dimensional images, allowing precise evaluation of the vertical, buccolingual, and anteroposterior positions of impacted teeth. This information enables a more comprehensive assessment of surgical complexity than that provided by conventional radiographs [5].

Recognizing the limitations of conventional radiographic assessments, Maglione et al. [6] proposed a CBCT-based classification to evaluate the three-dimensional relationship between impacted mandibular third molars and the inferior alveolar canal. This classification enhanced preoperative risk assessment for inferior alveolar nerve injury and improved consistency in communication among clinicians. Although this classification represents an important advancement in CBCT-based assessment, it primarily focuses on the anatomical relationship with the mandibular canal and does not objectively quantify three-dimensional impaction depth or evaluate its ability to predict overall surgical difficulty, including operative time, bone removal, and the need for tooth sectioning. Therefore, there remains a need for an objective, quantitative, and clinically applicable three-dimensional index that integrates the spatial depth of impacted mandibular third molars and predicts surgical difficulty more accurately than conventional classification systems.

Accordingly, the present study was designed to investigate the association between the three-dimensional impaction depth of mandibular third molars and surgical difficulty using CBCT. To address the limitations of existing assessment methods, we developed a novel three-dimensional impaction depth index (3D-IDI) that integrates the vertical, buccolingual, and anteroposterior dimensions into a single quantitative measure. The primary objective was to determine the predictive ability of the 3D-IDI in identifying surgically difficult mandibular third molars and to establish a clinically useful cut-off value. The secondary objectives were to evaluate the relationship between the 3D-IDI and operative time, surgeon-assigned difficulty score, and the requirement for tooth sectioning, as well as to identify the independent CBCT-derived predictors of operative time. We hypothesized that greater three-dimensional impaction depth would be independently associated with increased surgical difficulty and that the proposed 3D-IDI would accurately predict surgically difficult cases.

## Materials and methods

Study design and setting

This prospective observational study was conducted at the Department of Oral and Maxillofacial Surgery, Bharati Vidyapeeth (Deemed to be University) Dental College and Hospital, Sangli, Maharashtra, India, over a period of 12 months from October 2022 to September 2023. The study included patients requiring surgical removal of the unilateral impacted mandibular third molars who fulfilled the predefined eligibility criteria. All participants underwent preoperative clinical examination and CBCT imaging before the surgical intervention. The study protocol was designed to evaluate the influence of three-dimensional impaction depth on the surgical difficulty encountered during mandibular third molar removal.

Ethical approval

Approval for this investigation was granted by the Institutional Ethics Committee of Bharati Vidyapeeth (Deemed to be University) Dental College and Hospital, Sangli (approval no. BVDU/DCH/09/2022-23; September 12, 2022), before the initiation of the study. Before participation, all individuals were provided with comprehensive information regarding the purpose and study procedures, and written informed consent was obtained from each participant. The research was carried out in compliance with the ethical standards of the Declaration of Helsinki and its later revisions.

Sample size estimation

The required sample size was estimated a priori using G*Power software (version 3.1.9.7; Heinrich Heine University, Düsseldorf, Germany). A two-tailed point-biserial correlation model was selected to detect the association between the 3D-IDI and surgical difficulty. A moderate effect size (r = 0.28) was obtained from a previous study, and by using an alpha level of 0.05 and a statistical power of 80%, the minimum required sample size was calculated as 82 impacted teeth [1]. Considering a potential 10% loss due to incomplete CBCT measurements or procedural data, the final sample size was increased to 90 unilaterally impacted mandibular third molars.

Eligibility criteria

The eligibility criteria are summarized in Table 1.

**Table 1:** Inclusion and exclusion criteria CBCT: cone-beam computed tomography

Inclusion criteria	Exclusion criteria
1. Systemically healthy patients	1. Patients with acute infection associated with impacted tooth
2. Age between 18 and 40 years	2. Patients with odontogenic cysts or tumors
3. Presence of unilateral impacted mandibular third molar	3. Patients with craniofacial anomalies
4. Impacted mandibular third molar requiring surgical removal	4. Patients with previous surgical intervention in the third molar region
5. Preoperative CBCT images of adequate diagnostic quality	5. Patients with systemic diseases affecting bone metabolism
6. Preoperative CBCT images suitable for accurate three-dimensional measurements	6. Patients with systemic diseases affecting wound healing
7. Patient willingness to participate in the study	7. Patients receiving medications known to influence bone turnover
8. Only one impacted mandibular third molar per participant (to ensure independence of observations)	8. Patients with poor-quality CBCT images unsuitable for accurate three-dimensional measurements

Study methodology

**Figure 1:**
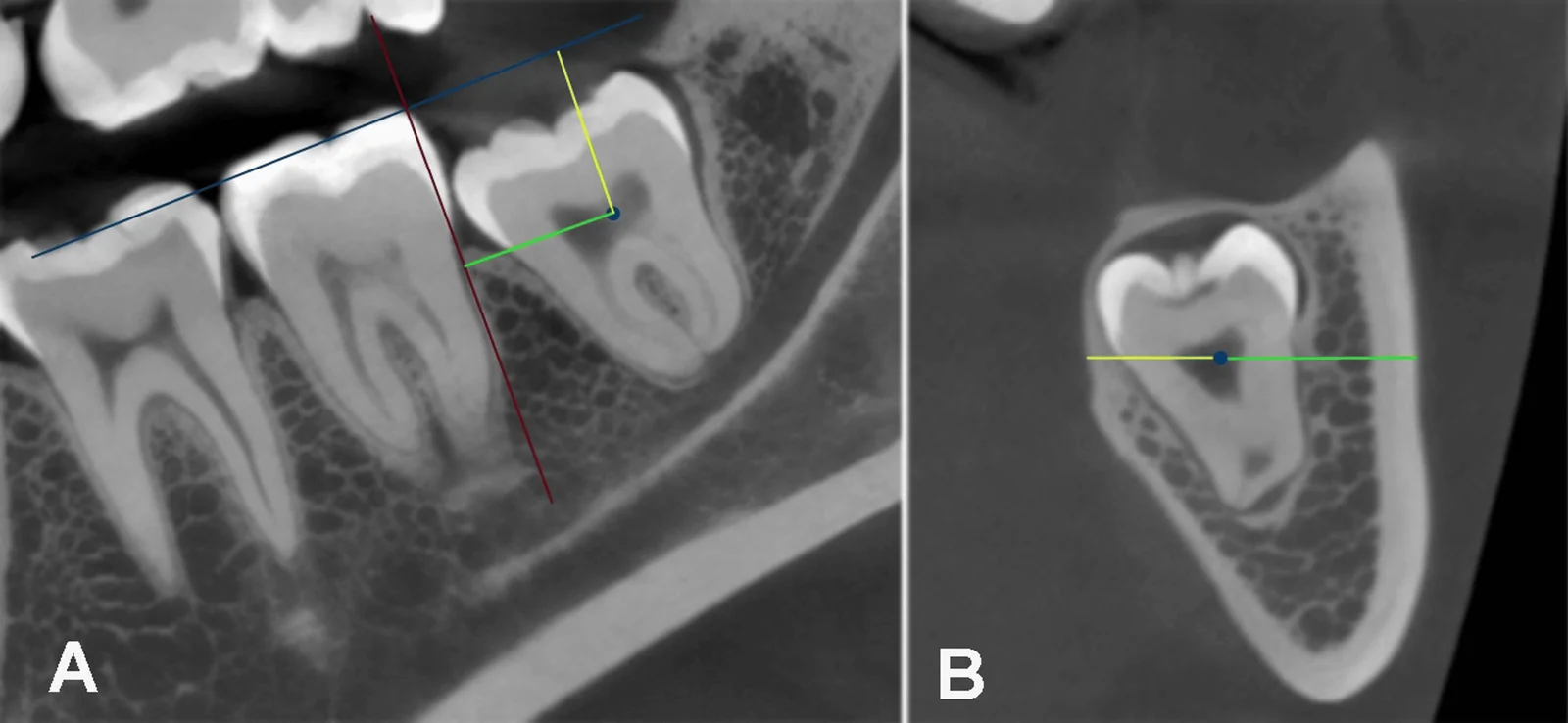
Cone-beam CT image of impacted mandibular third molar The image shows (A) anteroposterior length (green line) and vertical depth (yellow line) in sagittal view, (B) buccal (green line) and lingual (yellow line) width in axial view Blue line: occlusal plane; red line: distal surface of second mandibular molar; blue dot: central reference point on impacted mandibular third molar CT: computed tomography

The 3D-IDI is a novel index developed by the authors for this study and has not been previously described or adapted from any published classification or index. Following recruitment, all participants underwent standardized preoperative CBCT examinations using a Carestream CS 9600 CBCT system (Carestream Dental LLC, 3625 Cumberland Boulevard SE, Suite 700, Atlanta, GA). Standardized three-dimensional measurements were obtained from the preoperative CBCT images after image orientation had been standardized in the axial, coronal, and sagittal planes to ensure consistent anatomical alignment and measurement reproducibility. Vertical depth (V) was measured on the sagittal section as the perpendicular distance from the occlusal plane, defined by a line joining the cusps of the adjacent mandibular molars, to the highest point of the impacted third molar. Buccolingual width (BL) was measured on the coronal section as the maximum horizontal distance between the buccal and lingual cortical plates passing through the center of the impacted tooth. Anteroposterior length (AP) was determined on the axial section as the horizontal distance along the dental arch from the distal surface of the adjacent second molar to the center (or mesial surface) of the impacted third molar (Figure 1).

All measurements were performed by a trained examiner to ensure consistency, and the mean of two readings was recorded for each parameter to minimize measurement error.

The 3D-IDI was subsequently calculated using the Euclidean distance equation:

\[ \mathrm{3D\mbox{-}IDI}=\sqrt{V^{2}+BL^{2}+AP^{2}} \]

where V represents vertical depth, BL represents buccolingual width, and AP represents anteroposterior length. The calculated index was retained as a continuous variable for the subsequent predictive and correlation analyses. The Euclidean distance equation was selected because it provides a mathematically robust method for combining three mutually orthogonal CBCT measurements into a single quantitative index representing the overall three-dimensional spatial position of the impacted tooth. Equal weighting of the vertical, buccolingual, and anteroposterior components was intentionally adopted because no validated evidence currently supports assigning greater importance to any individual dimension. A weighted model was therefore not employed to avoid introducing arbitrary assumptions and to maintain mathematical simplicity, reproducibility, and clinical applicability. Future studies may evaluate regression-derived or machine learning-based weighting schemes to determine whether differential weighting further improves predictive performance.

All surgical extractions were carried out under local anesthesia according to a standardized operative protocol. After elevation of a full-thickness mucoperiosteal flap, osteotomy was performed using a rotary surgical handpiece under copious sterile saline irrigation to prevent thermal injury. When required, the impacted tooth was sectioned to facilitate controlled and minimally traumatic extraction. To ensure procedural consistency and eliminate inter-operator variability, all surgeries were performed by the same experienced oral and maxillofacial surgeon (SS). Operative time was measured in minutes, beginning with the initial mucosal incision and ending with placement of the final suture. Upon completion of the procedure, the operating surgeon independently rated the overall surgical difficulty on a 10-point numerical rating scale (0-10), with higher scores representing greater operative complexity [7].

Calibration and reliability testing

Before commencement of the study, two independent specialists (AR, KG), who were not affiliated with Bharati Vidyapeeth (Deemed to be University) Dental College and Hospital, Sangli, served as blinded examiners for radiographic measurements. Both examiners were unaware of the study objectives, patient identity, clinical findings, and surgical outcomes during the measurement process. Before the formal assessment, the examiners underwent a calibration session using CBCT scans that were not included in the final study sample to standardize the measurement protocols for vertical, buccolingual, and anteroposterior depths.

After calibration, each examiner independently evaluated 20 randomly selected CBCT scans under identical viewing conditions. To determine intra-examiner reliability, the same CBCT scans were re-evaluated by each examiner after a two-week interval without access to the initial measurements. Reliability was assessed using the two-way random-effects, absolute-agreement, single-measure intraclass correlation coefficient (ICC (2,1)). The inter-examiner ICC was 0.89 (95% confidence interval (CI): 0.75-0.95), while the intra-examiner ICC values were 0.91 (95% CI: 0.80-0.96) for examiner 1 and 0.88 (95% CI: 0.73-0.95) for examiner 2, indicating excellent measurement reliability and reproducibility of the CBCT-derived measurements.

Statistical analysis

The collected data were entered into Microsoft Excel and analyzed using IBM SPSS Statistics for Windows (version 25.0; IBM Corp., Armonk, NY). The normality of continuous variables was evaluated using the Shapiro-Wilk test. Normally distributed variables were expressed as mean ± standard deviation and compared using the independent t-test, whereas non-normally distributed variables were expressed as median and interquartile range and compared using the Mann-Whitney U test. Categorical variables are presented as frequencies and percentages and were analyzed using the chi-square test. Receiver operating characteristic (ROC) analysis was performed to evaluate the predictive ability of 3D-IDI for identifying surgically difficult cases, and the optimal cut-off value was determined using the Youden index.

Correlations between CBCT-derived measurements and surgical difficulty parameters were assessed using Spearman's rank correlation coefficient. Multivariable linear regression analysis was subsequently performed to identify independent predictors of operative time, while adjusting for potential confounding variables. Multicollinearity was assessed using tolerance and variance inflation factor (VIF). All predictor variables demonstrated tolerance values > 0.20 and VIF values < 5.0, indicating the absence of significant multicollinearity. All statistical tests were two-tailed, and statistical significance was set at p < 0.05.

## Results

Ninety patients with unilateral impacted mandibular third molars were included in this study. The mean age of the participants was 27.13 ± 4.56 years, and 49 (54.4%) were females. The mean 3D-IDI was 11.04 ± 2.68 mm, with 51 (56.7%) of cases classified as having high surgical difficulty (Table 2).

**Table 2:** Descriptive characteristics of the study population SD: standard deviation; 3D-IDI: three-dimensional impaction depth index

Variable		Value (N = 90)
Age (years)	Mean ± SD	27.13 ± 4.56
	Range	18–38
Sex, n (%)	Male	41 (45.6)
	Female	49 (54.4)
Vertical depth (mm)	Mean ± SD	7.50 ± 2.08
Buccolingual width (mm)	Mean ± SD	5.49 ± 1.52
Anteroposterior length (mm)	Mean ± SD	5.81 ± 1.55
3D-IDI score (mm)	Mean ± SD	11.04 ± 2.68
Difficulty category, n (%)	Low difficulty (score ≤ 5)	39 (43.3)
	High difficulty (score > 5)	51 (56.7)
Duration of surgery (min)	Mean ± SD	42.13 ± 14.10

ROC curve analysis demonstrated excellent predictive performance of 3D-IDI for identifying surgically difficult cases, with an area under the curve (AUC) of 0.938 (95% CI: 0.888-0.988; p < 0.001). The optimal cut-off value was 9.67 mm, which yielded a sensitivity of 88.2%, specificity of 89.7%, and an overall diagnostic accuracy of 88.9% (Table 3).

**Table 3:** ROC curve analysis of the 3D-IDI for predicting high surgical difficulty ROC curve analysis was performed to determine the optimal cut-off value using the Youden index ROC: receiver operating characteristic; 3D-IDI: three-dimensional impaction depth; AUC: area under the curve; CI: confidence interval

Parameter	Value
AUC	0.938
95% CI (Hanley–McNeil)	0.888–0.988
Standard error	0.026
z statistic (vs. AUC = 0.5)	17.18
p-value	< 0.001
Optimal cut-off (Youden index)	9.67 mm
Youden index (J)	0.780
Sensitivity at cut-off	88.2%
Specificity at cut-off	89.7%
Positive predictive value	91.8%
Negative predictive value	85.4%
Overall accuracy	88.9%

A comparison between the deep (> 9.67 mm) and shallow (< 9.67 mm) impaction groups revealed no significant differences in age or sex distribution (p > 0.05). However, the vertical, buccolingual, and anteroposterior depths, as well as the 3D-IDI score, were significantly greater in the deep impaction group (all p < 0.001) (Table 4).

**Table 4:** Demographic and CBCT characteristics according to 3D-IDI ^*^Statistical significance was set at p < 0.05 Continuous variables were compared using the independent-samples t-test or Mann–Whitney U test, and categorical variables using the chi-square test CBCT: cone-beam computed tomography, 3D-IDI: three-dimensional impaction depth; SD: standard deviation; IQR: interquartile range

Variable		Deep, > 9.67 mm (n = 49)	Shallow, < 9.67 mm (n = 41)	Test statistic	P-value
Age (years)	Mean ± SD	27.76 ± 4.35	26.39 ± 4.74	t = 1.423	0.158
Sex, n (%)	Male	19 (38.8)	22 (53.7)	χ² = 1.994	0.158
	Female	30 (61.2)	19 (46.3)		
Vertical depth (mm)	Mean ± SD	9.05 ± 1.34	5.64 ± 0.97	t = 13.564	< 0.001^*^
Buccolingual width (mm)	Mean ± SD	6.47 ± 1.30	4.31 ± 0.74	t = 9.383	< 0.001^*^
Anteroposterior length (mm)	Mean ± SD	6.78 ± 1.29	4.65 ± 0.88	t = 8.968	< 0.001^*^
3D-IDI (mm)	Median (IQR)	13.55 (11.15–14.62)	8.75 (8.11–9.14)	U = 2009	< 0.001^*^

Patients with deep impactions exhibited significantly longer operative time, higher surgical difficulty scores, and a greater frequency of tooth sectioning than those with shallow impactions (all p ≤ 0.002) (Table 5).

**Table 5:** Comparison of surgical difficulty parameters according to 3D-IDI ^*^Statistical significance was set at p < 0.05 Continuous variables were compared using the independent-samples t-test or Mann–Whitney U test, and categorical variables using the chi-square test 3D-IDI: three-dimensional impaction depth; SD: standard deviation; IQR: interquartile range

Variable		Deep (n = 49)	Shallow (n = 41)	Test statistic	P-value
Operative time (min)	Mean ± SD	42.45 ± 4.93	30.02 ± 4.02	t = 12.930	< 0.001^*^
Difficulty score (0–10)	Median (IQR)	7 (6–8)	4 (4–5)	U = 1908	< 0.001^*^
Surgical difficulty, n (%)	More difficult	45 (91.8)	6 (14.6)	χ² = 54.182	< 0.001^*^
	Less difficult	4 (8.2)	35 (85.4)		
Tooth sectioning required, n (%)	Yes	34 (69.4)	15 (36.6)	χ² = 9.684	0.002^*^
	No	15 (30.6)	26 (63.4)		

Spearman's correlation analysis demonstrated significant positive correlations between all CBCT-derived depth measurements and surgical difficulty parameters. 3D-IDI showed the strongest positive correlation with operative time (ρ = 0.901) followed by difficulty score (ρ = 0.860) (Table 6).

**Table 6:** Correlation between CBCT-derived impaction depth measurements and surgical difficulty parameters ^*^Statistical significance was set at p < 0.05 Correlations (ρ) were assessed using Spearman's rank correlation test CBCT: cone-beam computed tomography, 3D-IDI: three-dimensional impaction depth

CBCT parameter	Operative time		Difficulty score	
	ρ	P-value	ρ	P-value
3D-IDI (mm)	0.901	< 0.001^*^	0.860	< 0.001^*^
Vertical depth (mm)	0.863	< 0.001^*^	0.818	< 0.001^*^
Buccolingual width (mm)	0.715	< 0.001^*^	0.723	< 0.001^*^
Anteroposterior length (mm)	0.798	< 0.001^*^	0.759	< 0.001^*^

Multivariable linear regression analysis identified vertical depth (β = 0.508; p < 0.001) and anteroposterior depth (β = 0.326; p < 0.001) as significant independent predictors of operative time, whereas age, sex, and buccolingual width were not. The overall regression model explained 81.9% of the variance in the operative time (adjusted R² = 0.808; p < 0.001) (Table 7).

**Table 7:** Multivariable linear regression analysis of predictors of operative time ^*^Statistical significance was set at p < 0.05 Multivariable linear regression analysis was performed to identify independent predictors of operative time. Regression coefficients are presented as unstandardized coefficient (B), standard error (SE), standardized coefficient (β), 95% confidence interval (CI), and p-value. Model fit: R² = 0.819, adjusted R² = 0.808, F(5,84) = 75.80, p < 0.001

Predictor	B	SE	β	t value	95% CI of coefficient	P-value
Constant	8.486	2.782	—	3.050	2.953–14.018	0.003^*^
Age (years)	0.053	0.082	0.031	0.642	−0.110–0.215	0.522
Sex (male vs. female)	−1.197	0.769	−0.078	−1.556	−2.727–0.333	0.123
Vertical depth (mm)	1.884	0.273	0.508	6.894	1.340–2.427	< 0.001^*^
Buccolingual width (mm)	0.710	0.361	0.141	1.965	−0.009–1.429	0.053
Anteroposterior length (mm)	1.620	0.372	0.326	4.359	0.881–2.359	< 0.001^*^

## Discussion

The present prospective observational study evaluated the influence of three-dimensional impaction depth, assessed using CBCT, on the surgical difficulty of impacted mandibular third molar removal. To objectively quantify three-dimensional impaction depth, we developed a novel three-dimensional impaction depth index (3D-IDI), which integrates the vertical, buccolingual, and anteroposterior dimensions into a single quantitative measure. The findings demonstrated that the proposed 3D-IDI was strongly associated with both objective and subjective indicators of surgical difficulty. Greater three-dimensional impaction depth was associated with longer operative time, increased bone removal, a higher requirement for tooth sectioning, and greater surgeon-perceived difficulty. Moreover, the 3D-IDI exhibited excellent diagnostic performance for predicting surgically difficult cases, highlighting the value of a comprehensive three-dimensional assessment in improving preoperative estimation of surgical complexity. As this is the first study to introduce and evaluate the proposed 3D-IDI, further external validation in larger, independent populations is warranted before its widespread clinical implementation.

In the present study, the mean age of the participants was 27.13 years, with a slight predominance of female patients. This demographic distribution is consistent with previous epidemiological studies, which have reported that impacted mandibular third molars are most frequently managed during the second and third decades of life, when root development is complete and surgical removal is commonly indicated [8]. Similar age distributions have been reported by Glodha et al. [1], while other investigators have evaluated factors influencing third molar surgery [8,9].

One of the most important findings of this study was the excellent diagnostic performance of the 3D-IDI in predicting surgically difficult cases, with an area under the ROC curve of 0.938 and an optimal cutoff value of 9.67 mm. Although previous investigations have demonstrated that CBCT improves the visualization of impacted third molars and adjacent anatomical structures, most studies have relied on individual radiographic variables or conventional classification systems [10]. The present study extends existing knowledge by integrating vertical, buccolingual, and anteroposterior measurements into a single quantitative index. This approach provides a more comprehensive assessment of spatial tooth position and may explain the superior predictive performance observed.

Patients with deeper impactions experienced significantly longer operative times and greater surgical difficulties than those with shallow impactions [11]. Operative time is widely accepted as one of the most objective indicators of surgical complexity because it reflects the technical challenges encountered during surgery [12]. Similar observations have been reported by Renton et al. [12], Susarla and Dodson [13], Glodha et al. [1], and Bello et al. [14], who demonstrated that increasing impaction depth and unfavorable tooth position significantly prolonged surgical procedures and increased the risk of postoperative complications. However, unlike previous investigations that primarily relied on two-dimensional radiographic classifications, the present study objectively quantified three-dimensional depth using CBCT measurements, thereby providing a more precise assessment of surgical complexity.

The present findings are consistent with those reported by Vasegh et al. [15], who evaluated 340 mandibular third molars using CBCT and observed that the proximity of the roots to the inferior alveolar canal varied significantly according to tooth angulation. Specifically, the distance between the distal root and the inferior alveolar canal showed a significant association with Winter's angulation classification, highlighting the influence of impaction orientation on the anatomical relationship with the neurovascular bundle.

This study also demonstrated that deep impactions required significantly greater bone removal and more frequent tooth sectioning. These findings are clinically expected because teeth positioned deeper within the mandibular bone generally require wider osteotomy and additional sectioning to facilitate safe removal while minimizing excessive force [14]. Previous reports have similarly shown that increasing impaction depth is associated with more invasive surgical procedures, although most authors have evaluated depth qualitatively rather than quantitatively [15,16]. The use of a continuous three-dimensional measurement in the present study provides stronger evidence to support this relationship.

Correlation analysis revealed that the composite 3D-IDI demonstrated the strongest positive association with operative time, surgical difficulty score, and bone removal volume. Among the individual components, vertical depth was the strongest predictor of operative difficulty. Multivariable regression analysis further confirmed that vertical and anteroposterior depths independently influenced operative time, whereas buccolingual depth showed only a borderline association. These findings suggest that although all spatial dimensions contribute to surgical complexity, the depth of the tooth below the occlusal plane and its posterior position have the greatest influence on the extent of surgical intervention required [11]. This observation is consistent with previous studies that identified vertical depth as one of the principal determinants of surgical difficulty, although few studies have simultaneously evaluated all three orthogonal dimensions [16-18].

The findings of the present study differ from those of several earlier investigations that reported only moderate correlations between radiographic findings and surgical difficulty [16,18]. Such differences may be attributed to methodological variations, including differences in sample characteristics, imaging techniques, assessment criteria, surgeon experience, and the use of conventional two-dimensional radiographs instead of CBCT. Consistent with these observations, Akadiri and Obiechina [19], in a systematic review, identified impaction depth, angulation, root morphology, patient age, surgeon-related factors, and operative variables as the principal determinants of surgical difficulty, emphasizing that no single anatomical parameter can accurately predict operative complexity. Therefore, the incorporation of vertical, buccolingual, and anteroposterior components into the proposed 3D-IDI may explain the superior predictive performance observed in the present investigation.

From a clinical perspective, the proposed 3D-IDI may represent a valuable adjunct to preoperative treatment planning. Accurate prediction of surgical difficulty enables clinicians to allocate appropriate operating times, anticipate the need for advanced surgical techniques, counsel patients regarding procedural complexity and potential complications, and determine whether referral to an experienced oral and maxillofacial surgeon is warranted. The use of a quantitative CBCT-derived index may also contribute to greater standardization of surgical difficulty assessments in both clinical practice and future research.

The present study has certain limitations. The study was conducted at a single institution with a relatively modest sample size, which may limit the generalizability of the findings. Surgical difficulty was partially based on the surgeon’s assessment, which, despite the use of a standardized scoring system, may be influenced by operator experience. Additionally, postoperative outcomes, such as pain, swelling, trismus, and complications, were not evaluated. Furthermore, all surgical procedures were performed by a single experienced oral and maxillofacial surgeon. Although this approach minimized inter-operator variability and ensured a standardized surgical protocol, it may limit the generalizability of the findings to surgeons with different levels of clinical experience or varying surgical techniques. Future multicenter studies involving larger and more diverse populations, as well as multiple operators with varying levels of surgical experience, are warranted to validate the applicability and generalizability of the proposed 3D-IDI across different clinical settings and to further investigate its relationship with postoperative morbidity and long-term surgical outcomes.

## Conclusions

Within the limitations of this single-center study, the CBCT-derived 3D-IDI appears to be a reliable predictor of the surgical difficulty of impacted mandibular third molar removal. Greater three-dimensional impaction depth was significantly associated with longer operative time, a greater requirement for tooth sectioning, and higher surgical difficulty scores. The proposed quantitative index shows promise as an adjunct to preoperative risk assessment and surgical planning; however, its broader clinical applicability should be confirmed through external validation in larger, multicenter studies before routine clinical implementation.
